# High-resolution 3D visualization of ductular proliferation of bile duct ligation-induced liver fibrosis in rats using x-ray phase contrast computed tomography

**DOI:** 10.1038/s41598-017-03993-2

**Published:** 2017-06-26

**Authors:** Lili Qin, Xinyan Zhao, Jianbo Jian, Yuqing Zhao, Mengyu Sun, Chunhong Hu

**Affiliations:** 1 College of Biomedical Engineering, Tianjin Medical University, Tianjin, 300070 China; 2 Liver Research Center, Beijing Friendship Hospital, Capital Medical University, Beijing, 100050 China

## Abstract

X-ray phase-contrast computed tomography (PCCT) can provide excellent image contrast for soft tissues with small density differences, and it is particularly appropriate for three-dimensional (3D) visualization of accurate microstructures inside biological samples. In this study, the morphological structures of proliferative bile ductules (BDs) were visualized without contrast agents via PCCT with liver fibrosis samples induced by bile duct ligation (BDL) in rats. Adult male Sprague-Dawley rats were randomly divided into three groups: sham operation group, 2-week and 6-week post-BDL groups. All livers were removed after euthanasia for a subsequent imaging. The verification of the ductular structures captured by PCCT was achieved by a careful head-to-head comparison with their corresponding histological images. Our experimental results demonstrated that PCCT images corresponded very well to the proliferative BDs shown by histological staining using cytokeratin 19 (CK19). Furthermore, the 3D density of proliferative BDs increased with the progression of liver fibrosis. In addition, PCCT accurately revealed the architecture of proliferative BDs in a 3D fashion, including the ductular ramification, the elongation and tortuosity of the branches, and the corrugations of the luminal duct surface. Thus, the high-resolution PCCT technique can improve our understanding of the characteristics of ductular proliferation from a new 3D perspective.

## Introduction

Corresponding to the different types of liver injury, the dynamic and diverse reconstruction of the biliary architecture may represent an adaptive response of the tissue, and should have several physiological advantages^1^. Liver fibrosis induced by common bile duct ligation (BDL) represents an experimental model of human cholestatic liver fibrosis. The development and variation of cholestatic liver diseases correlate with changes in the ductular structure^2^. The biliary network of the liver is very important in the formation and secretion of bile and the excretion of toxic substances through the bile ductules (BDs). To explore the anatomical basis and remodelling process that occurs in response to intrahepatic cholestasis, the three-dimensional (3D) visualization of the biliary tree is essential. 3D reconstruction can yield structural information on the biliary tract architecture and enable quantitative anatomical studies^3, 4^.

While liver biopsy and the histological assessment of the liver play important roles in clinical management, it must be recognized that the liver biopsy has some inherent limitations such as sampling variability, sampling error and destructiveness^5^. At the same time, although clinical imaging techniques, including ultrasound, conventional radiography, computed tomography (CT) and magnetic resonance imaging (MRI), can be used to observe some serious pathological changes, the microstructures of the liver tissues are invisible to these techniques due to restrictions of their spatial resolution and contrast. Thus, there is a need for highly sensitive, reliable imaging techniques that enable physicians to accurately reconstruct the morphological structure of intrahepatic ductular proliferation and better understand the pathologies of liver fibrosis.

X-ray phase contrast imaging (PCI) has been demonstrated to be advantageous in revealing detailed structures inside biological specimens. Unlike conventional absorption-based x-ray imaging, the contrast in PCI arises from the phase shift that x-rays suffer when they travel through a sample. Generally, PCI has 1000-fold greater sensitivity than conventional absorption-based x-ray imaging. Combined with the advantages of PCI and CT, x-ray phase contrast CT (PCCT) has been developed. PCCT exhibits superior properties over conventional absorption-based CT, where biological samples show very weak absorption contrast. Thus, PCCT is very suitable for soft tissue evaluation with small density variations. In recent years, PCCT has been widely used in biological tissue imaging, including that of the kidney, hepatocellular carcinoma, hepatic alveolar echinococcosis, spinal cord, breast cancer and coronary atherosclerotic plaques^6–11^. This technique presents new possibilities for analysing and characterizing the anatomical and pathological features of diseased microvasculature in liver fibrosis. Combining PCCT with a 3D visualization technique, the 3D microvascular morphology of liver fibrosis can be clearly revealed, and qualitative descriptions and quantitative assessments of the microvasculatures demonstrate clear differences between the different stages of liver fibrosis^12–14^. In addition, our previous study confirmed that PCCT can clearly depict the microvascular structures from different stages of liver fibrosis induced by BDL without contrast agents, but accurate visualizations of proliferative BDs cannot be presented on account of the restriction of the charge coupled device (CCD) spatial resolution^15^.

The structural visualization of ductular proliferation may facilitate the study of pathological changes associated with cholestatic liver fibrosis induced by BDL. High-resolution 3D images of the morphological features of proliferative BDs can better provide insight into the pathology of ductular proliferation of liver fibrosis. In this study, liver fibrosis induced by BDL in rats was used to perform the experiment without contrast agents, and the proliferative BDs were visualized using PCCT. The purpose of this study was to observe 3D structures of proliferative BDs and explore the corresponding ductular reaction mechanisms and the development changes of proliferative BDs during the course of liver fibrosis induced by BDL using PCCT.

## Results

### PCCT image and 3D microvascular structures

**Figure 1:**
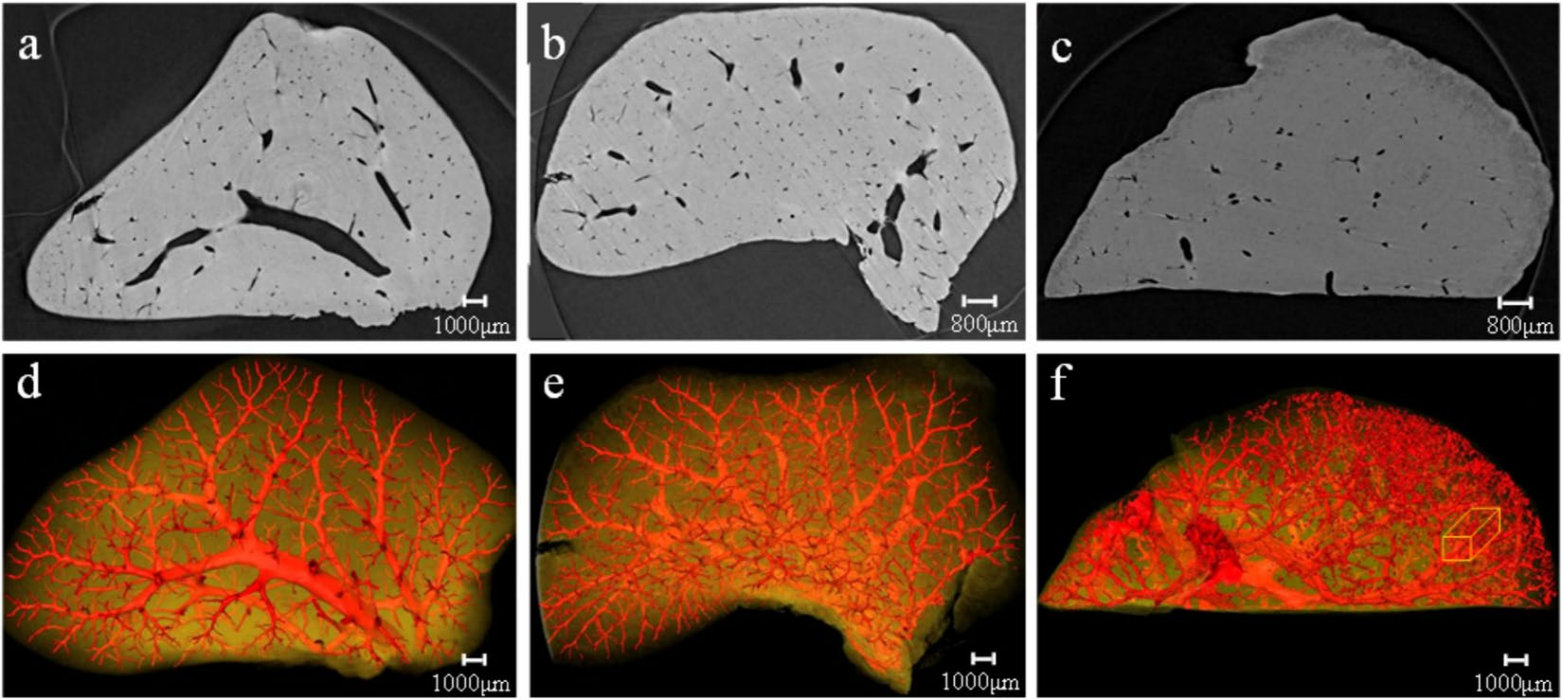
Slices and corresponding 3D microvasculature rendering images from different groups. A hepatic vascular tree is presented with regular shapes in the control group (**a**,**d**). Tortuous and abnormal microvascular structure caused by fibrosis tissues occured at week 2 post-BDL (**b**,**e**). The disordered microvascular structure and abnormal microvascular morphology become more apparent at week 6 post-BDL as the fibrosis stage increased (**c**,**f**). The ductular proliferation marked by a yellow cuboid is revealed in (**f**).

The CT image and 3D microvasculature for a liver sample in the control group are shown in Fig. 1(a,d), respectively. In Fig. 1d, the microvascular tree of the liver was clearly exhibited with regular shapes, dichotomous branches and stereoscopic effects. Similarly, the results at week 2 post-BDL (Fig. 1b,e) show that a few microvascular branches exhibited lightly irregular structures (Fig. 1e), and due to the limitation of the CCD spatial resolution, there is no obvious ductular proliferation. In contrast, the results at week 6 post-BDL are shown in Fig. 1(c,f), respectively. In Fig. 1f, the 3D volume rendering showed seriously disordered and abnormal microvascular structures. The irregular microvascular branches were clearly revealed, and some rough granular structures were also presented, as shown in the area of the yellow cuboid region. The following sections demonstrated that they were the result of ductular proliferation according to pathobiology. However, the proliferative BDs were disorganized without an apparent tubular structure. To observe the morphological characteristics of the proliferative BDs, the regions of ductular proliferation were selected as regions of interest (ROIs) for the next high-resolution imaging.

### PCCT imaging and histopathological analysis

**Figure 2:**
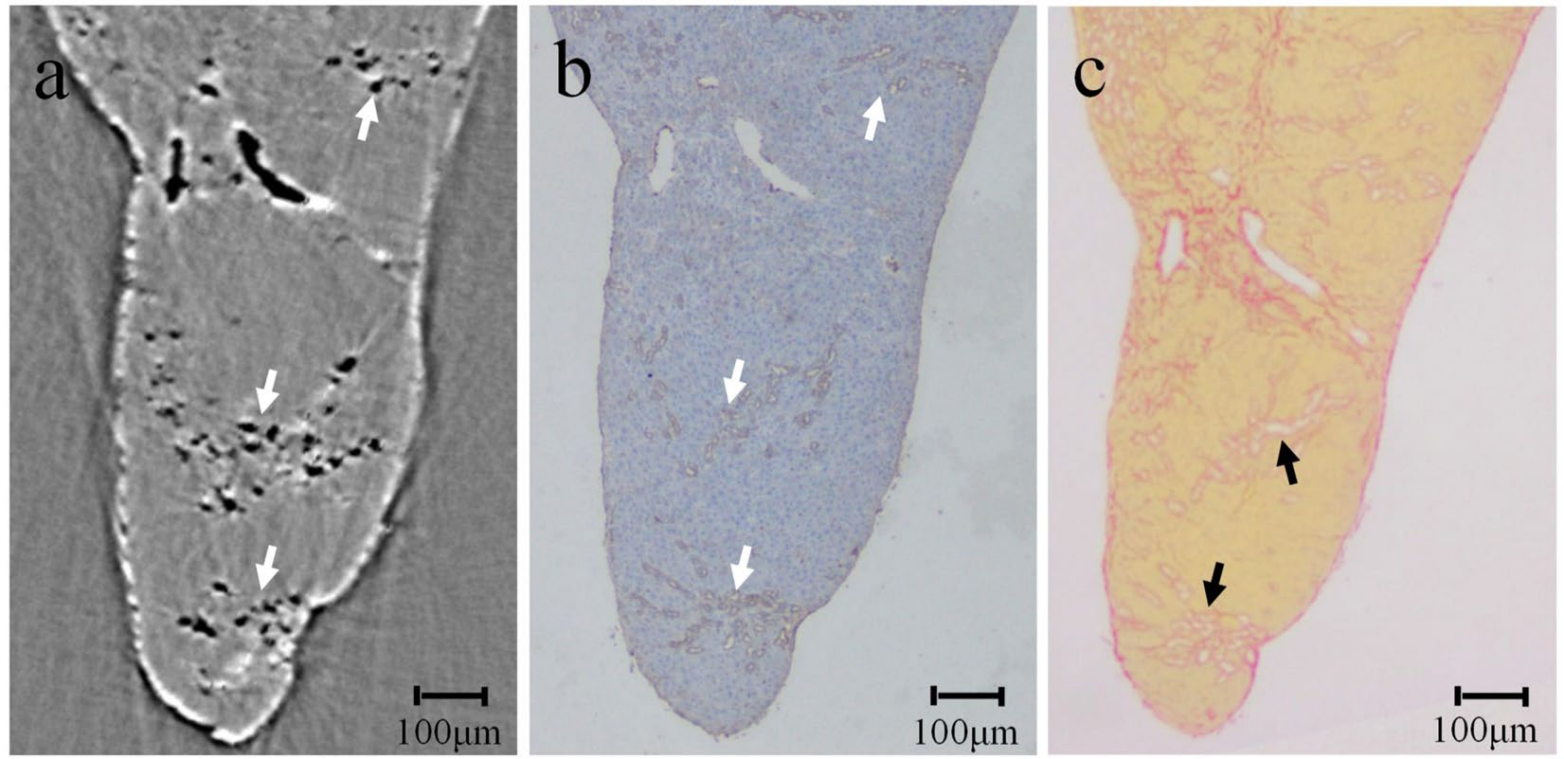
CT image and corresponding pathological sections of the BDL rats. In the immunohistochemical staining for CK19 (**b**), CK19-expressing cells appear to form larger clusters that are stained brown, which is in accordance with the CT findings (**a**), which are marked by the white arrows. In the histological slice stained with Sirius Red (**c**), fibrous bands are clearly shown within the regions marked by black arrows.

The PCCT image and corresponding histological sections of the 6-week post-BDL group are shown in Fig. 2. Abundant ductular proliferations, stained with cytokeratin 19 (CK19), are shown in Fig. 2b (brown colour), and these findings were in accordance with CT image listed in Fig. 2a, which revealed that the selected the ROIs in Fig. 1f were the regions of ductular proliferation. Clearly, PCCT was able to detect the architecture of the proliferative BDs. Additionally, a dense vascular plexus surrounded by fibrous bands can be clearly observed in Fig. 2c.

### Ductular proliferation in Liver Fibrosis Progression

**Figure 3:**
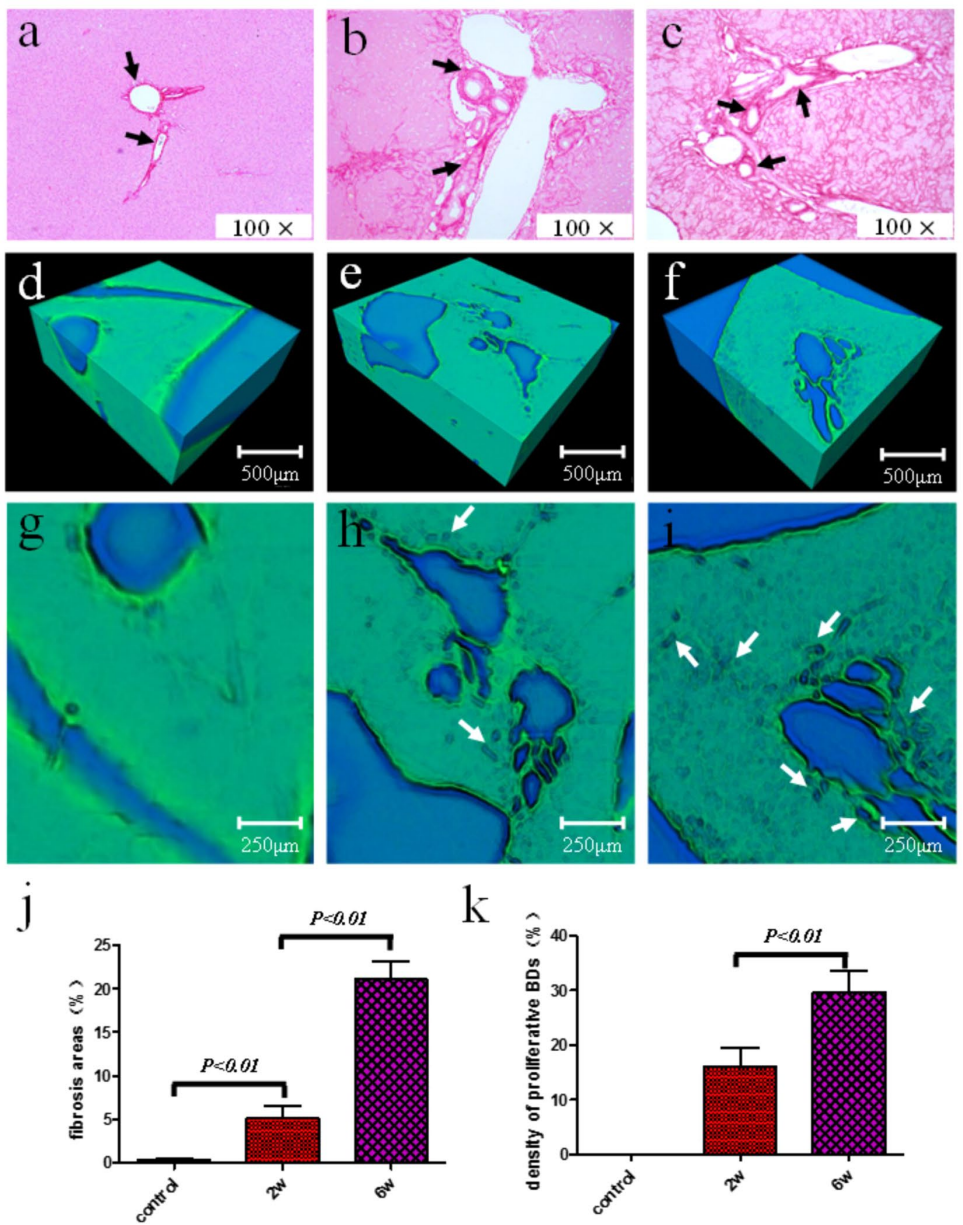
Illustration and quantification of fibrosis and proliferative BDs in liver fibrosis progression. Histological sections stained by Sirius Red showing representative images of the control group, the 2-week and 6-week post-BDL groups are shown in (**a**) to (**c**). The volume rendering images of three groups are shown in (**d**) to (**f**). The amplified images of the upper surface in (**d**), (**e**) and (**f**) are provided in (**g**) to (**i**), respectively. Fibrosis area (**j**) and the density of proliferative BDs (**k**) are summarized and shown for different groups. The black and white arrows indicate the fibrous bands and proliferative BDs, respectively. For all comparisons, *P* was less than 0.01. Note that the ductular proliferations are not detected in the livers of the control group.

The Histological sections stained for collagen with Sirius Red were used to assess the degree of fibrosis (Fig. 3a–c). In the livers of the control group, collagen was detected in low amounts around the portal tracts and hepatic veins (Fig. 3a) without ductular proliferation (Fig. 3d,g). At week 2 post-BDL, fibrotic septa were gradually formed and normal architecture of the liver was divided into pseudo-lobules by fibrotic septa (Fig. 3b), and ductular proliferation began forming around portal tracts (Fig. 3e,h). Subsequently, at week 6 post-BDL, fibrosis continuously accumulated and increased, and hepatic pseudo-lobules were further divided into smaller pseudo-lobules (Fig. 3c). Moreover, abundant ductular proliferations increased quickly (Fig. 3f,i). The ductular proliferations were marked by the white arrows in Fig. 3(h,i). The amount of collagen manifested by relative fibrous areas was used to assess the degree of fibrosis. Control group exhibited fibrosis areas of 0.29 ± 0.12%, and the fibrosis areas at week 2 and week 6 post-BDL were 5.09 ± 1.38% and 21.07 ± 2.08%, respectively (Fig. 3j). Obviously, the fibrosis areas increased with development of liver fibrosis, while there was a significant difference among the three groups (all *P* < 0.01). Quantitative changes in the ductular proliferation, which were characterized by the density of proliferative BDs, also showed obvious changes during the progression of liver fibrosis, as shown in Fig. 3k. Compared with the 2-week post-BDL group (16.06 ± 3.39%), the density of proliferative BDs was 1.84-fold higher at week 6 post-BDL (29.63 ± 3.98%). The density of proliferative BDs increased with increasing degree of liver fibrosis, and significant differences were found between the 2-week and 6-week post-BDL groups (*P* < 0.01).

### 3D ductular proliferation visualization

**Figure 4:**
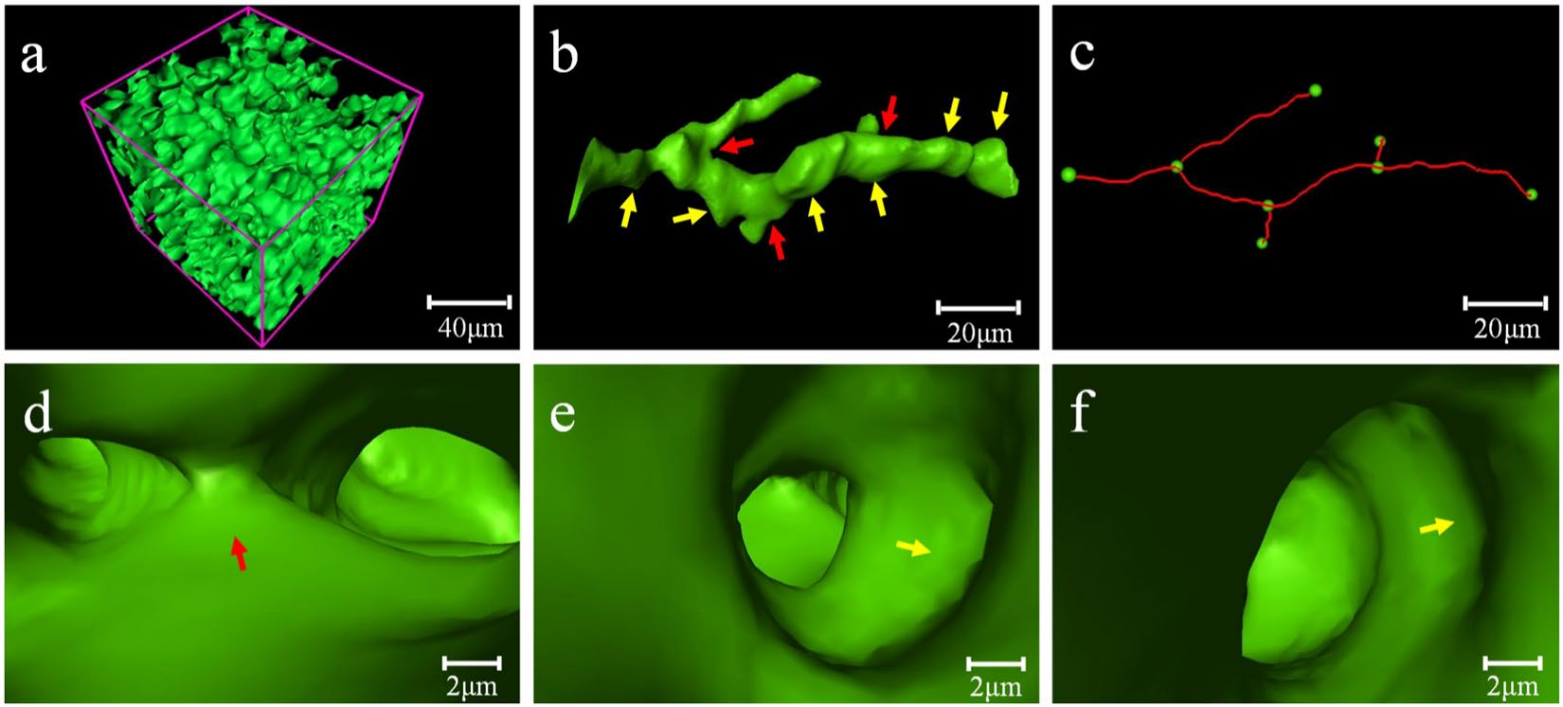
3D reconstruction of ductular proliferation and VME to 3D track targeted BD. (**a**) Local 3D reconstruction of proliferative BDs. (**b**) A single segmented BD. Bifurcations in (**b)** and (**d**) (red arrows), and the corrugations in (**b**), (**e**) and (**f**) (yellow arrows) are discernible. (**c**) Centre line of the segmented BD in (**b**). The branching points and breakpoints are marked by green dots. Successive pathway tracing is shown in (**d**) to (**f**).

The local 3D reconstruction of the ductular proliferation regions, which is marked by the white arrows in Fig. 2a, is clearly depicted in Fig. 4a. Dense proliferative BDs were well represented (see supplementary Video S1). To further observe the architectures and bifurcations of the BDs, a single BD was segmented (Fig. 4b). A BD with a minimum diameter of approximately 10 μm was detected, and duct elongation, and branching were clearly visualized. The red arrows indicated bifurcations in Fig. 4(b,d). The proliferative BDs showed a relatively uneven, wrinkled luminal bile duct surface (namely, corrugations), which were marked by yellow arrows in Fig. 4(b,e,f). In Fig. 4c, the centre line of the segmented BD was extracted, and the pattern of biliary branches was also clearly presented. It can be observed that the shape of the centre line was tortuous, and the bile ductular branches were dichotomous, which was the same as the bifurcated arrangement of the biliary tree in the normal liver^16^. The 3D microstructural features of the targeted bile ductular lumen can be identified (Fig. 4d–f) using the virtual micro-endoscopy (VME) technique, and the endobiliary bifurcations are clearly discernible in Fig. 4d. In addition, corrugations were formed through the evagination of a two dimensional (2D) planar tissue surface into 3D (Fig. 4e,f), and this evagination could also be observed in the 2D CT images and histological sections obtained from the liver samples of the rats and the human biliary liver cirrhosis (see supplementary Fig. S1 and S2). 3D virtual technology with automatic navigation through the bile ductular lumen could provide a powerful tool for the stereoscopic visualization of the endobiliary space. Successive pathway tracing images were presented in the supplementary materials (see supplementary Fig. S3), and a virtual endoscope video was also provided in supplementary Video S2.

## Discussion

Our preliminary experimental data provided evidence that high-resolution 3D imaging by PCCT can accurately detect the microstructural morphological features of proliferative BDs in BDL rats without injecting contrast agents. A comparison of the CT images with the histological sections of the samples highlighted the high degree of sensitivity of the PCCT technique as a non-invasive imaging modality. In contrast, histological sections provide only 2D images of the microvasculatures, and 3D morphological analysis of the microvasculature is very difficult to obtain. Moreover, histological sections destroy the tissue integrity, and have the complex sample preparation process and limited vision for analysis^5^. In practice, the microscopy techniques, such as scanning electron microscopy (SEM), transmission electron microscopy (TEM), and light microscopy (LM), can provide extraordinary spatial resolutions to micron and nanometre scale, but they are limited to surface or very small depth (∼tens of micrometres) information^17–19^. Recently, the confocal microscopy study of the cholestasis-induced 3D-architectural changes of interlobular bile ducts has recently become available, and the connections between the bile duct and the lobular bile canalicular network by the canals of Hering were clearly presented, but this method needs immunohistochemical staining for serial microtome sections, and has weak penetration power to reveal the internal structure of the liver tissue^2^. Two-photon microscopy can also present 3D microvascular structure at the microscopic level, but it is limited by the imaging depth^20^. Imaging techniques, such as CT, MRI, and magnetic resonance angiography (MRA), have inherent advantages in the direct visualization of anatomical structure of hepatic vessels. Unfortunately, the vascular changes at the micron or submicron levels are invisible to current imaging techniques due to insufficient contrast and spatial resolution^21, 22^. Micro-CT, depending on the absorption-contrast imaging, has been used as a visualization tool for the microstructures of the samples at high resolutions, but soft tissues imaging are typically impossible via micro-CT without the use of contrast agents because of small differences in attenuation values^22^. A previous study showed that PCCT could obtain high depiction of microstructural details and soft tissue contrast as well as higher contrast-to-noise ratios than absorption-based micro-CT^23^. Compared with other modalities, PCCT provides superior soft tissues contrast without any contrast agent, which will inflict less damage to the biological tissue^24, 25^.

Recently, PCI has made great progress in liver fibrosis imaging. It has been demonstrated that PCI can detect blood vessels, the fibrotic septa, fibrotic hepatic lobules and dilated bile ducts in the livers of rodents without the use of any contrast agent. However, projection overlapping reduces the accuracy of the imaging because surface features may obscure the internal structures of the liver^26–28^. The studies have shown that blood vessels down to the micrometre level can be clearly revealed by PCCT without contrast agents, and high-resolution 3D microvasculature was presented based on experimental liver fibrosis in rodents induced by thioacetamide, albumin immune complex and BDL^12, 14, 15^. Clear visualization and accurate analysis of microvascular changes can characterize different stages of fibrosis progression. In previous study, PCCT well characterized the anatomical properties and pathological features of the microvasculature from different stages of liver fibrosis induced by BDL, and differentiated between types of blood vessels and BDs^15^. However, because of the limitation of the CCD spatial resolution, it is not adequate for the accurate microstructural observation of ductular proliferation. With the increase of the spatial resolution, the 3D high-resolution PCCT imaging technique can well overcome these limitations. In this study, accurate 3D microvascular structures of the liver were reconstructed using PCCT, and they were presented in a disordered state due to ductular proliferation. Furthermore, with 3D reconstruction, the ductular proliferation could be more directly observed. High-resolution 3D visualization can be utilized not only to reconstruct the outline of proliferative BDs but also to observe the corresponding micron-sized structural changes. Moreover, the study showed that the density of proliferative BDs increased with development of liver fibrosis. Thus, PCCT presents a new method for analysing and characterizing the anatomical and pathological features of the ductular proliferation, and this technique can provide useful information for the diagnosis of cholestatic liver fibrosis.

Transporters and enzymes that are involved in the adaptive remodelling triggered by the BDL are present on the luminal surface of the BD, and there is an increase in the functional capacity of the biliary tree to reabsorb bile salts and alleviate cholestasis as the intraluminal surface area increases^29, 30^. The adaptive remodelling induced by BDL aims at optimizing the intraluminal surface area by corrugation, duct branching and branch elongation, and such structures may cause an expansion of the absorptive surface area. In the adaptive modification process, the proliferation of the cholangiocytes is a typical pathological performance according to sections stained with CK19. It is apparent that a substantial amount of cholangiocyte proliferation is directed towards the formation of ductular branches, elongation and corrugations. The tortuosity of the BD may be caused by the cholestatic stress and collagen deposition. These corresponding microstructural changes could result in the disproportionate increase in surface area, similar to the function of villi in the intestine or sulci in the brain, where an expansion of absorptive and cortical surface area is achieved, respectively^2^. In this study, PCCT accurately revealed the morphological features of the proliferative BDs by 3D imaging. The clear visualization of the proliferative BDs allowed us to confirm that ductular proliferation could cause ductular ramification, the elongation and tortuosity of the branches, and the corrugation of the luminal duct surface. In addition, VME was utilized to track the pathway of a targeted BD by the 3D visualization technique, and it enabled the visualization of the microstructures of the bile ductular lumen and inner walls, including the corrugations and bifurcations. VME provided a novel method for the visualization of the endobiliary space on the basis of 3D reconstruction and revealed altered inner ductular architecture in the ductular proliferation. Hence, 3D external imaging and endoscopic tracking will make it possible to accurately observe the micromorphological characteristics of BDs in the research on cholangiopathies.

The experiments were performed by using the excised *ex vivo* samples with a synchrotron set-up. While used with *ex vivo* tissues, PCCT could be used for nondestructive presentation of the microvascular structures in liver fibrosis compared with traditional histology, and thereby contribute complementary information to histopathologic findings, such as detailed 3D microvascular morphologic characteristics. Moreover, PCCT includes a wider view of the liver instead of a small sample in liver biopsy. Although not yet applicable *in vivo*, PCCT has the potential to become a valuable tool to monitor liver fibrosis process noninvasively. Further studies need to be performed *in vivo* to investigate the PCCT value in liver fibrosis analysis. The *in vivo* visualization of intrahepatic BDs will be our new direction in follow-up studies. In principle, PCCT can be performed for *in vivo* imaging with acceptable or less doses to the sample by using optimized design of experimental instrument and new reconstruction CT algorithms^8, 31, 32^. While *in vivo* imaging for liver fibrosis using PCCT is still challenging due to motion artifacts and overlaying structures, some preliminary *in vivo* biomedical studies have recently focused on the demonstration of the high diagnostic significance of PCI or PCCT techniques^33, 34^. It is worth noting that application to a wider range of clinical and biomedical studies awaits the development of higher-performance laboratorial x-ray sources. Actually, PCI techniques on a conventional x-ray tube have been developed^35^, the 3D structural analyses of biliary diseases that include clinical specimens bode well for the widespread practical application of PCCT methods in the near future. *In vivo* assessment of liver fibrosis might be achievable without the use of contrast agents when PCCT becomes available in clinical scanners. Additionally, PCCT could provide insights into the 3D alteration of ductular proliferation in this study. To further understand the adaptive remodelling induced by BDL, more studies need to focus on the observation of dynamic changes of the proliferative BDs in progressive liver fibrosis, and a large number of samples need to be used to demonstrate statistical significance. The subsequent studies will be carried out using a higher number of samples and groups based on different time points. In this study, the imaging of the canalicular network and canals of Hering is still limited by the resolution compared to liver biopsy and confocal microscopy. PCCT has sufficient resolution to detect microvessels on micrometer or submicrometer scales, and thus the further improved spatial resolution can overcome this limitation.

In summary, the preliminary experimental results of this study demonstrated that PCCT could detect the microstructural features of ductular proliferation in BDL rats. The ductular ramification, elongation and tortuosity of branches, and corrugations of the luminal duct surface were clearly presented. The clear 3D visualization of the proliferative BDs, without the use of a contrast medium, could be a promising method to evaluate the characteristics of proliferative BDs. In the wake of developments in PCCT, this technique may play an auxiliary role in monitoring the ductular proliferation of chronic liver diseases.

## Methods

### Sample preparation

All animal experiments were carried out in accordance with the guiding principles for the care and use of laboratory animals approved by the Research Ethics Committee of Beijing Friendship Hospital, Capital Medical University. Fifteen male Sprague-Dawley rats (8 weeks old, weighing 180–220 g) were used in the study. The rats were kept in a constant temperature environment, where food and tap water were available ad libitum. All rats were randomly divided into the following three groups: sham operation group, 2-week and 6-week post-BDL groups. At week 2 (5 rats) and week 6 (5 rats) post-BDL, the ligation of the common bile duct (CBD) was performed to induce biliary fibrosis. In brief, under phenobarbital anaesthesia, the CBD was isolated, double-ligated using 4.0 silk threads and cut in between after a midline abdominal incision. Subsequently, rat livers were harvested at week 2 and week 6 post-BDL. The sham operation group (5 rats) acted as a control group, in which the CBD underwent laparotomy but no ligation. All the liver samples were immersed in 10% buffered formalin before imaging.

To further explore the feasibilities in clinical practice of PCCT, the sample of human biliary liver cirrhosis from one patient was used to perform the supplementary experimental validation. The human liver sample was provided by the Beijing Friendship Hospital, Capital Medical University, and written informed consent was obtained from the patient. The patient (female, 7 years old) had been diagnosed with biliary cirrhosis and underwent liver resection. The liver cirrhosis tissue was gathered within 24 hours after liver resections and was fixed in neutral phosphate-buffered formalin (10%).

### Image acquisition

**Figure 5:**
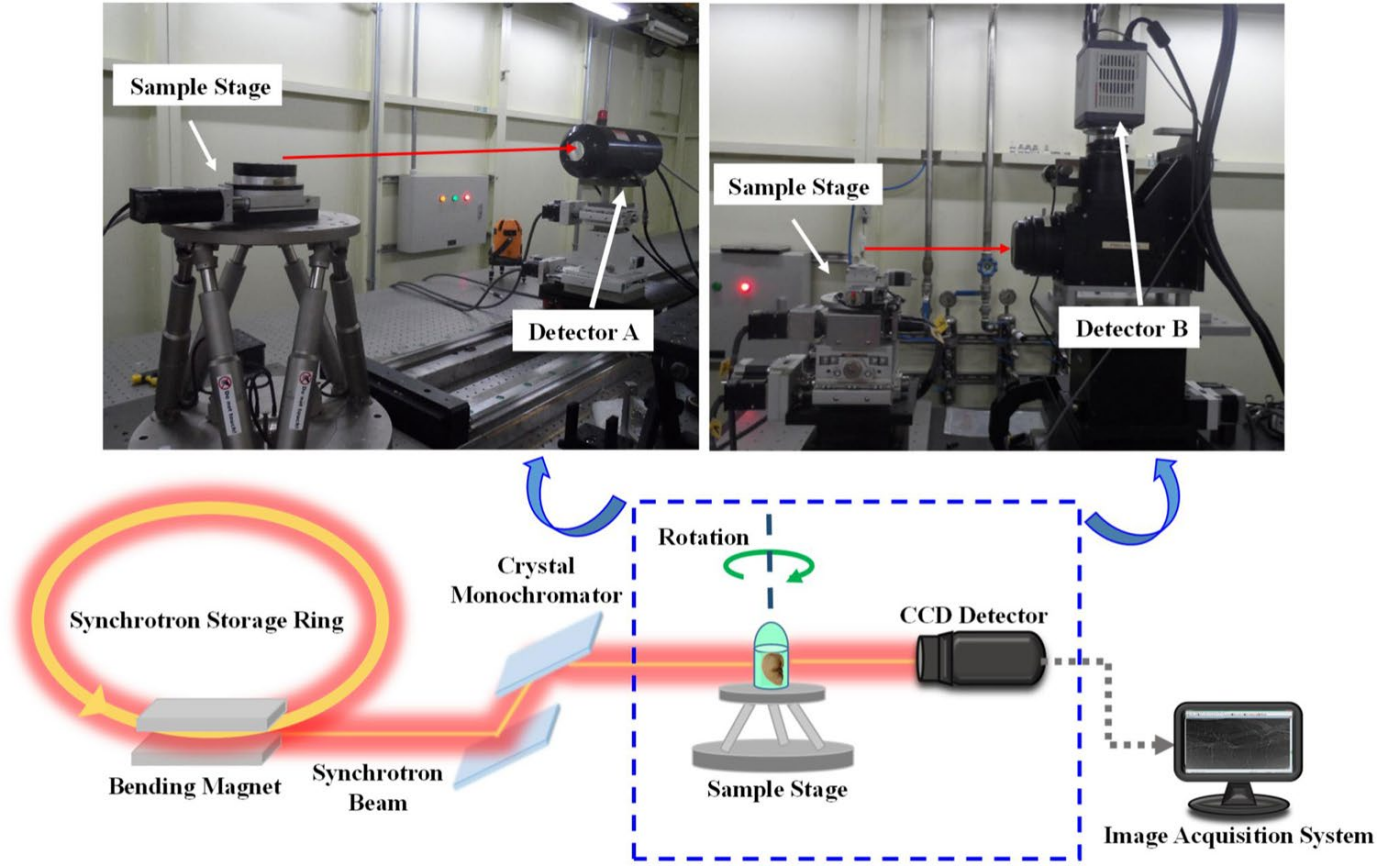
Schematic illustration of the experimental station at BL13W1. A monochromatized x-ray is projected on a liver sample that was mounted on a rotary stage. The scanning images can be acquired at a proper distance by adjusting the SDD that can be varied from 0.05 m to a maximum of 8 m. In this study, the transmitted beam was recorded by two different image detectors with spatial resolution of 9 μm (detector A) and 3.25 μm (detector B), respectively. During the CT imaging, the projections were collected over 180° of rotation.

PCCT was employed to capture images of the samples on the x-ray imaging and biomedical application beamline (BL13W1) at the Shanghai Synchrotron Radiation Facility (SSRF) in China. X-rays were produced from a 3.5 GeV electron storage ring, and the tuneable energy range of the x-ray beam was 8–72.5 keV. The samples were placed 34 m from the synchrotron source, and the distance between the sample and the detector could be adjusted from 0 cm to 8 m. A thin (100 μm) CdWO4 cleaved single-crystal scintillator and a CCD detector were used to obtain images. The schematic of the experimental setup is shown in Fig. 5. In the imaging system, the incident white synchrotron x-ray beam emerging from the accelerator was monochromatized by a double-crystal monochromator. By setting a proper sample-detector distance (SDD), the object was illuminated by the highly parallel and monochromatic x-ray beam, and the transmitted beam was measured by a detector.

In this study, the x-ray energy was adjusted to 24 keV, the exposure time was set to 12 ms per projection, and the SDD was adjusted to 80 cm. As the sample stage rotated over 180°, a total of 733 initial projection images were captured. In addition, 10 dark images (dark signal in the absence of photons) and 10 flat images (with no sample in the beam) were used to perform a dark-field correction and a flat-field correction, respectively^36^. The total time of scanning was approximately 25 min. The x-rays transmitted through the object were detected by the CCD camera with a matrix size of 4008 × 2672, a pixel size of 9 × 9 μm^2^, and the field of view was approximately 36 mm × 5 mm. The liver was positioned perpendicular to the plane of the X-ray beam. Stack images were then utilized to reconstruct the CT slices using the filtered back projection algorithm. Finally, 3D reconstructions were performed using Amira software (Visage Imaging, Berlin, Germany) and the 3D morphology of the microvasculature could be observed from different views.

For better visualization of the ductular proliferation, ROIs of the samples, which included ductular proliferations, were selected to obtain high-resolution images at BL13W1. Similarly, the scanned parameters were optimized and set to conditions with a photon energy of 14 keV, a matrix size of 2048 × 2048 and a pixel size of 3.25 × 3.25 μm^2^. The field of view was 6.656 mm × 5 mm and the SDD was adjusted to 30 cm for PCCT. The sample was mounted at the sample stage and rotated 180° around its central axis, resulting in 720 projections. The exposure time per projection was 1.2 s. During the exposure period, 8 flat field images were collected for every 240 projections, and 5 dark field images were obtained and used to reduce the dark current noise from the projections. The total time of the x-ray exposure was approximately 30 min. The 3D reconstructions of the BDs were also performed using Amira, and the morphological characteristics of the proliferative BDs in the BDL group were observed. The flow chart of the process of making images is provided in the supplementary materials (see supplementary Fig. S4).

### Image Analysis

After the imaging studies, the liver samples were routinely processed, embedded in paraffin, and cut into 4-μm-thick slides. All slides were stained with Sirius Red and immunostained with antibodies against CK19 for the detection of collagen and biliary epithelial cells, respectively. The histological sections were prepared at the planes where the CT images were acquired, and its corresponding analysis was performed by an experienced pathologist (X. Z., with more than 10 years of experience in liver pathology). The histological findings served as the reference for the interpretation of the CT images. The fibrosis area, stained with Sirius Red, was expressed as a relative percentage between the area of fibrosis and the total sample area, and it was used to evaluate the degree of liver fibrosis^37^. Twenty-five non-overlapping slides from each group at 100× magnification were assessed. The fibrosis area was analyzed using Image-Pro Plus 6.0 software (Media Cybernetics, Bethesda, Maryland, USA). The quantitative changes in the ductular proliferation during the development of fibrosis can be evaluated by analysis of the 3D density of proliferative BDs. Twenty-five cubic volumes of interest (VOIs) of the ductular proliferation regions from each group were selected, with each VOI consisting of 50 × 50 × 50 voxels (as shown in Fig. 4a). The percentage taken by the total bile ductular volume within the VOI is calculated out of the total volume of the VOI based on the 3D reconstruction^38^.

### Virtual bile-duct micro-endoscopy

This study further attempted to perform VME on the basis of 3D reconstruction to investigate the inner microstructures of proliferative BDs. Generate Surface Model had been generated and exported as a Surface View Model in Amira. The procedures of image processing included path planning and real-time rendering^39^. Pressing the seek button and then clicking on an arbitrary object in the scene caused the object to be moved into the centre of the viewer window. Moreover, the camera was oriented parallel to the normal direction at the selected point. “Virtual tracing” was realized for the inner visualization of the targeted BD by changing the position and orientation of the camera.

### Statistical analyses

All statistical analysis was performed using the SPSS software package (version 20; IBM, Chicago, USA). The data are reported as the the means ± standard deviations. *P* values were calculated using paired Student t-test to assess statistical differences. A *P* value of less than 0.05 was considered to be statistically significant.

## Electronic supplementary material

Supplementary Materials

Supplementary Video S1

Supplementary Video S2
