# Supplementary material for: High-resolution 3D visualization of ductular proliferation of bile duct ligation-induced liver fibrosis in rats using x-ray phase contrast computed tomography
Source: Sci Rep. 2017 Jun 26;7:4215. doi: 10.1038/s41598-017-03993-2 (PMC5484700; doi:10.1038/s41598-017-03993-2)
Supplement: Supplementary file 1 — Supplementary Materials [file 41598_2017_3993_MOESM1_ESM.pdf]

High-resolution 3D visualization of ductular proliferation of bile duct ligation-induced liver fibrosis in rats using x-ray phase contrast computed tomography

Lili Qin[1], Xinyan Zhao[2], Jianbo Jian[1], Yuqing Zhao[1], Mengyu Sun[1] & Chunhong Hu[1]

[1]College of Biomedical Engineering, Tianjin Medical University, Tianjin 300070, China. [2]Liver Research Center, Beijing Friendship Hospital, Capital Medical University, Beijing 100050, China.

Supplementary Information

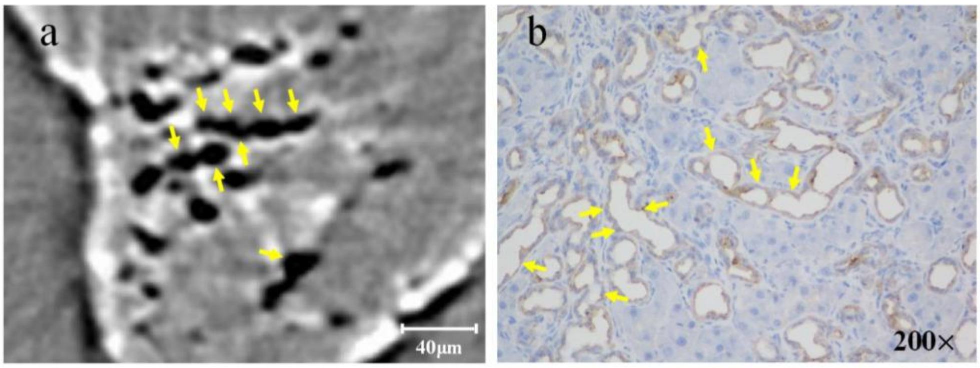

Supplementary Fig. S1 CT image and pathological sections of the BDL rats. In the CT image (**a**) and immunohistochemical staining for CK19 (**b**), the presence of corrugations on the luminal bile duct surface is observed (yellow arrows). The high-resolution CT image has a close resemblance to the optical images of the stained histological section.

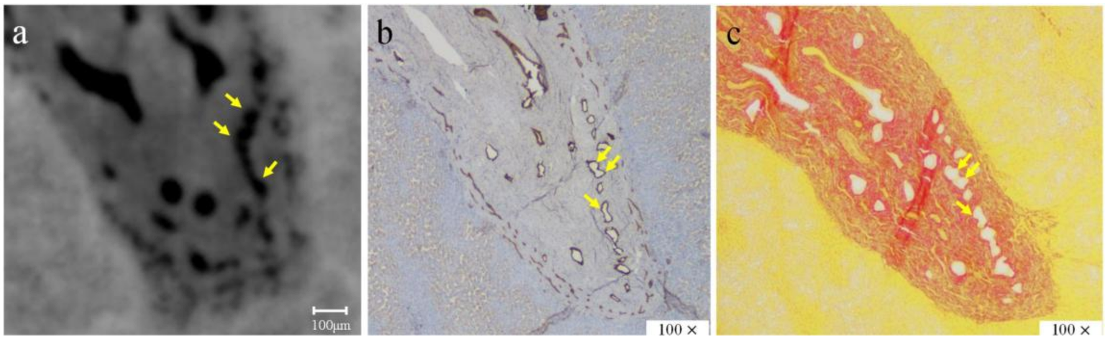

Supplementary Fig. S2 CT image and corresponding pathological sections in human biliary liver cirrhosis. (**a**) PCCT image of the human liver sample. The corresponding histopathologic sections were stained with CK19 (**b**) and Sirius Red (**c**). The yellow arrows indicate the presence of corrugations on the luminal bile duct surface.

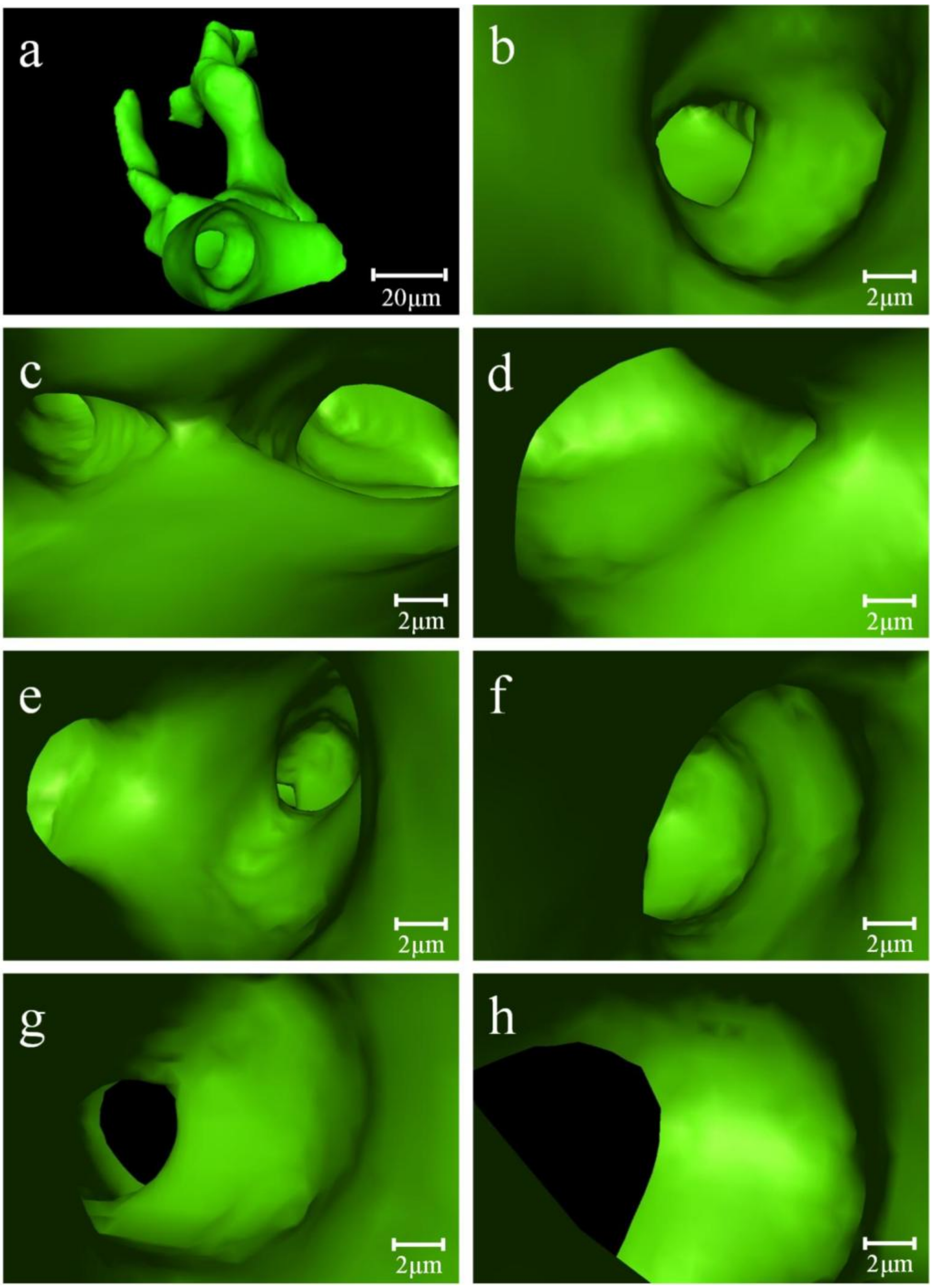

Supplementary Fig. S3 Virtual micro-endoscopy to 3D track targeted BD. (**a**) Initiation to orientate a BD. Successive pathway tracing is shown in (**b**) to (**h**). The endobiliary micro-structure is clearly discernible.

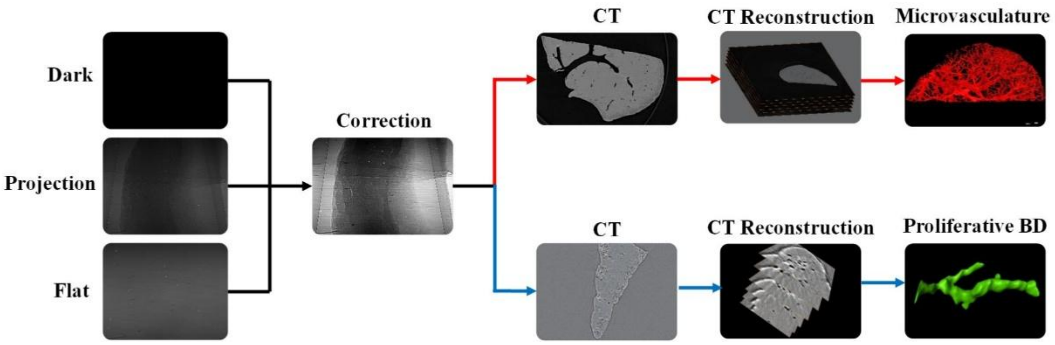

Fig. S4 Image processing for 3D reconstruction of intrahepatic microvasculature and the proliferative BD. The red arrows indicate reconstruction procedure of the intrahepatic microvasculature. The reconstruction procedure of the proliferative BD is well demonstrated by blue arrows.

Supplementary Movie S1 Animated view of the 3D structures of ductal proliferation. This is the same 3D model shown in Fig. 3a. The rotation of the model permits the viewers to better observe proliferative BD structures.

Supplementary Movie S2 Animated view of the 3D structures of segmented bile duct. This is the same 3D model shown in Fig. 3b. The 3D model can be rotated in real time to facilitate a clear visualization of the BD. The targeted BD is traced so that the inner of that can be clearly presented.
